# *FAM13A* and *POM121C* are candidate genes for fasting insulin: functional follow-up analysis of a genome-wide association study

**DOI:** 10.1007/s00125-018-4572-8

**Published:** 2018-02-27

**Authors:** Veroniqa Lundbäck, Agne Kulyte, Rona J. Strawbridge, Mikael Ryden, Peter Arner, Claude Marcus, Ingrid Dahlman

**Affiliations:** 1 Department of Clinical Science, Intervention and Technology, Division of Paediatrics, Huddinge, Karolinska Institutet, Stockholm, Sweden; 2 Department of Medicine, Huddinge, Karolinska Institutet, C2:94, SE-141 86 Stockholm, Sweden; 3 Institute of Health and Wellbeing, University of Glasgow, Glasgow, UK; 4 Department of Medicine, Solna, Karolinska Institute, Stockholm, Sweden

**Keywords:** Genomics, Insulin sensitivity, Lipid metabolism

## Abstract

**Aims/hypothesis:**

By genome-wide association meta-analysis, 17 genetic loci associated with fasting serum insulin (FSI), a marker of systemic insulin resistance, have been identified. To define potential culprit genes in these loci, in a cross-sectional study we analysed white adipose tissue (WAT) expression of 120 genes in these loci in relation to systemic and adipose tissue variables, and functionally evaluated genes demonstrating genotype-specific expression in WAT (eQTLs).

**Methods:**

Abdominal subcutaneous adipose tissue biopsies were obtained from 114 women. Basal lipolytic activity was measured as glycerol release from adipose tissue explants. Adipocytes were isolated and insulin-stimulated incorporation of radiolabelled glucose into lipids was used to quantify adipocyte insulin sensitivity. Small interfering RNA-mediated knockout in human mesenchymal stem cells was used for functional evaluation of genes.

**Results:**

Adipose expression of 48 of the studied candidate genes associated significantly with FSI, whereas expression of 24, 17 and 2 genes, respectively, associated with adipocyte insulin sensitivity, lipolysis and/or WAT morphology (i.e. fat cell size relative to total body fat mass). Four genetic loci contained eQTLs. In one chromosome 4 locus (rs3822072), the FSI-increasing allele associated with lower *FAM13A* expression and *FAM13A* expression associated with a beneficial metabolic profile including decreased WAT lipolysis (regression coefficient, *R* = −0.50, *p* = 5.6 × 10^−7^). Knockdown of *FAM13A* increased lipolysis by ~1.5-fold and the expression of *LIPE* (encoding hormone-sensitive lipase, a rate-limiting enzyme in lipolysis). At the chromosome 7 locus (rs1167800), the FSI-increasing allele associated with lower *POM121C* expression. Consistent with an insulin-sensitising function, *POM121C* expression associated with systemic insulin sensitivity (*R* = −0.22, *p* = 2.0 × 10^−2^), adipocyte insulin sensitivity (*R* = 0.28, *p* = 3.4 × 10^−3^) and adipose hyperplasia (*R* = −0.29, *p* = 2.6 × 10^−2^). *POM121C* knockdown decreased expression of all adipocyte-specific markers by 25–50%, suggesting that *POM121C* is necessary for adipogenesis.

**Conclusions/interpretation:**

Gene expression and adipocyte functional studies support the notion that *FAM13A* and *POM121C* control adipocyte lipolysis and adipogenesis, respectively, and might thereby be involved in genetic control of systemic insulin sensitivity.

**Electronic supplementary material:**

The online version of this article (10.1007/s00125-018-4572-8) contains peer-reviewed but unedited supplementary material, which is available to authorised users.

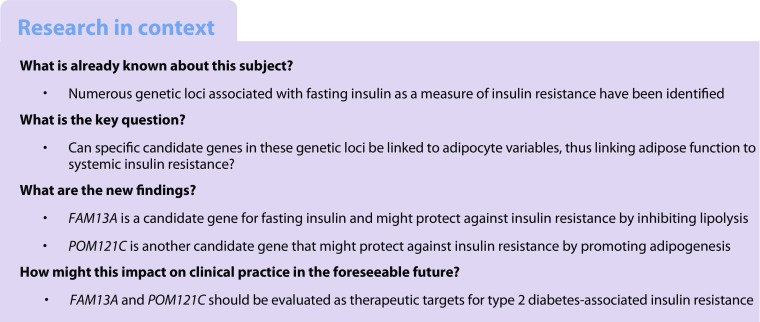



## Introduction

Insulin resistance (IR), wherein cellular responses to insulin are impaired, is a key component of type 2 diabetes and is also implicated in the development of cardiovascular disease [1]. IR is associated with metabolic disturbances in liver, skeletal muscle and white adipose tissue (WAT) and is characterised by hyperinsulinaemia, hyperglycaemia and dyslipidaemia. Fasting serum insulin (FSI) has been shown to correlate with the gold standard for assessing IR, namely euglycaemic insulin clamp, and is therefore used as a simple proxy for insulin sensitivity in various studies [2].

Abdominal obesity and a state of overweight are strong risk factors for systemic IR [3]. Abdominal obesity is associated with altered cytokine and adipokine release from WAT, which is linked to low-grade inflammation and systemic IR [4]. In addition, adipose morphology is central to IR and development of type 2 diabetes [5, 6]. WAT can expand by increasing the number and/or volume of adipocytes causing distinct adipose morphologies termed hyperplasia (many small adipocytes) or hypertrophy (few large adipocytes). Hypertrophic adipose tissue is associated with a pernicious metabolic profile, with blunted ability of insulin to stimulate fat synthesis through glucose conversion into lipids (lipogenesis), leading to an influx of NEFA into the liver and to systemic IR [7, 8]. The lipolytic activity in adipose tissue, resulting in the release of NEFA, can also influence insulin sensitivity, as reviewed [9].

Despite these findings, it is still to a large extent unclear how IR develops and only about 25% of obese women are insulin resistant [10]. One explanation is varying genetic predisposition; genome-wide association studies (GWAS) have identified 17 SNPs associated with FSI and/or FSI adjusted for BMI (FSIadjBMI) [11]. With the aim of defining culprit genes in these loci, and the mechanisms by which they may directly predispose to IR, we herein analysed WAT expression of 120 genes in these loci in relation to FSI and adipocyte phenotypes related to IR (morphology, insulin-stimulated lipogenesis, lipolysis). We also performed in silico expression quantitative trait locus (eQTL) analysis and selected genes demonstrating genotype-specific expression in WAT for functional analysis by small interfering RNA (siRNA) knockdown followed by evaluation of adipocyte-specific genes and glycerol release.

## Methods

### Participants

The study included 114 Swedish non-diabetic women with WAT global transcriptome profile available from a previous study [12]. The women were recruited by advertisement from the general adult population in the Stockholm (Sweden) area (Table 1). They displayed a large inter-individual variation in BMI and were healthy, except that some were obese. The study was approved by the regional ethics board in Stockholm and written informed consent was obtained from each participant. The experiments conformed to the principles set out in the WMA Declaration of Helsinki and the Department of Health and Human Services Belmont Report. Participants were investigated at 08:00 hours after an overnight fast in a university clinic. Anthropometric measurements (height, weight, waist and hip circumference, blood pressure) were performed followed by venous blood sampling. WHR adjusted for BMI (WHRadjBMI) was calculated in a sex-specific manner by inverse-normal transformation of the residuals of the linear regression model: WHR adjusted for age, age^2^ and BMI [13]. Fasting plasma glucose (FPG) and lipids were analysed at the hospital’s routine chemistry laboratory. Plasma insulin was measured by ELISA (Mercodia, Uppsala, Sweden) as previously described [14]. The Mercodia Diabetes Antigen Control (10-1134-01/10-1164-01) was included as control in all ELISA runs; samples were visually inspected before each run and no sample showed signs of haemolysis.

**Table 1:** Characteristics of 114 examined women

Characteristic	Means±SD
Age, years	43 ± 11
BMI, kg/m^2^	34 ± 9
FPG, mmol/l	5.17 ± 0.65
FSI, pmol/l	63 ± 50
HOMA-IR	0.96 ± 2.12
Fasting plasma total cholesterol, mmol/l	4.9 ± 0.9
Fasting plasma HDL-cholesterol, mmol/l	1.4 ± 0.4
Fasting plasma triacylglycerols, mmol/l	1.3 ± 0.8
Fat cell volume, pl	731 ± 266
WAT morphology^a^, pl^b^	17 ± 165
Basal lipolysis^c^, μmol glycerol (2 h)^−1^ (10^7^ adipocytes)^−1^	4.26 ± 2.62
Insulin-stimulated lipogenesis^d^, nmol of glucose (2 h)^−1^ (10^7^ adipocytes)^−1^	4.71 ± 6.22

^a^Data were missing from 11 women

^b^Defined in the ‘WAT experiments’ section of the Methods

^c^Data were missing from 22 women

^d^Data were missing from five women

Following the clinical examination, an abdominal subcutaneous WAT biopsy was obtained by needle aspiration, as described [15]. All WAT samples were rapidly rinsed in sodium chloride (9 mg/ml) and specimens of 300 mg unfractionated WAT were immediately frozen in liquid nitrogen for subsequent RNA isolation. Remaining tissue was used immediately for cell culture experiments as described below. No follow-up of participants was performed.

### WAT experiments

The adipose tissue was brought to the laboratory, rinsed repeatedly in saline (154 mmol/l NaCl) and visible blood vessels and cell debris were removed. Adipose tissue specimens (about 1 g) were divided into portions, one of which was treated with collagenase to obtain isolated adipocytes as described [16]. The mean weight and volume of these cells were determined as previously described [17, 18]. A curve fit of the relationship between mean adipocyte volume and total fat mass was performed as previously reported to assess adipose tissue morphology [19]. The difference between the measured and the expected fat cell volume obtained from the mean curve fit at the corresponding fat mass determines adipose morphology. If the measured fat cell volume is larger than expected, adipose hypertrophy prevails, whereas the opposite is valid for hyperplasia. These values, which can be quantitatively assessed, were obtained from the calculations made previously [19].

Spontaneous unstimulated lipolytic activity was determined in adipose tissue explants as described [20]. In brief, pieces of adipose tissue (200 or 300 mg) were incubated for 2 h (100 mg/ml) at 37°C with air as the gas phase in Krebs–Ringer phosphate buffer (pH 7.4) supplemented with glucose (8.6 mmol/l), ascorbic acid (0.1 mg/ml) and bovine serum albumin (20 mg/ml). Glycerol release into the medium was measured using a sensitive bioluminescence method and expressed as amount of glycerol released per 2 h and 10^7^ adipocytes.

Adipocyte lipogenesis was determined as described [21]. In brief, isolated adipocytes were incubated in vitro in an albumin-containing buffer with [^3^H]glucose (5 × 10^5^ dpm/ml), unlabelled glucose (0.001 mmol/l) and varying concentrations (0–70 nmol/l) of human insulin (I 2643, Sigma-Aldrich, Stockholm, Sweden). The incubations were conducted for 2 h at 37°C with air as the gas phase. Incubations were stopped by rapidly chilling the incubation vials to 4°C. Thereafter, incorporation of radiolabelled glucose into adipocyte lipids was determined; this reflects lipogenesis and was expressed as the amount of glucose incorporated per adipocyte number, as described previously [21]. Values at the maximum effective insulin concentration are reported.

### Microarray data

Global transcriptome profiles of WAT from the clinical cohort were assessed by Gene 1.0 or 1.1 ST Affymetrix arrays and has been reported earlier [12]. In this study we limited the analysis to candidate genes for FSI or FSIadjBMI listed in Scott et al [11], as well as genes ±500 Kb of the tag-SNPs. Genome information was extracted from SNPPER (http://snpper.chip.org/bio/snpper-enter) using genome build 38 (accessed 9 August 2016). Ingenuity Pathway Analysis (https://www.qiagenbioinformatics.com/) was used for network analysis (accessed 18 September 2017). SNPnexus (http://snp-nexus.org/) was used for SNP annotation (accessed 10 June 2017) [22]. GTEx database (https://www.gtexportal.org/home/) was used to identify WAT eQTLs (accessed 28 August 2016).

### Adipocyte cell culture and small interfering RNA transfection

Isolation, growth and differentiation of human mesenchymal stem cells (hMSCs) were carried out as previously described [23]. hMSCs at day 4 of differentiation were transfected using a Neon electroporator (Invitrogen, Carlsbad, CA, USA) according to the manufacturer’s protocol. Briefly, 1,000,000 hMSCs were mixed with 40 nmol/l ON-TARGETplus SMARTpool small interfering RNAs (siRNAs) targeting *POM121C*, *UHRF1BP1*, *SNRPC* and *FAM13A* or non-targeting siRNA pool (Dharmacon, Lafayette, CO, USA) and electroporated using 100 μl Neon electroporation tip. Electroporation conditions were 1600 V, 20 ms width, 1 pulse. Electroporation was repeated until the required number of cells was collected for a certain experimental set-up. Following electroporation, the cells were plated in antibiotic-free medium at a density of 220,000 cells/well in 24-well plates. Medium was replaced 24 h post-transfection. The cells were cultured until day 7 or 12 of differentiation, at which time the medium and RNA were collected. hMSCs were also reverse transfected 24 h before induction of adipogenesis using ON-TARGETplus SMARTpool siRNAs targeting *FAM13A* or non-targeting siRNA pool (Dharmacon) as previously described [24].

### Quantitative RT-PCR

WAT specimens (100 mg) from the clinical samples were disrupted mechanically and RNA isolated using the RNeasy kit (Qiagen, Manchester, UK) according to the manufacturer’s instructions. The hMSCs were collected on days 7 and 12 after the induction of differentiation for isolation of RNA. Total RNA from the hMSCs cultures was extracted using NucleoSpin RNA II kit (Macherey-Nagel, Düren, Germany). The concentration and purity of RNA were measured using a Nanodrop ND-1000 Spectrophotometer (Thermo Fisher Scientific, Waltham, MA, USA). Reverse transcription was performed using the iScript cDNA synthesis kit (Qiagen) and random hexamer primers (Invitrogen). Quantitative RT-PCR was performed using commercial TaqMan probes (Thermo Fisher Scientific). Gene expression in the clinical cohort was normalised to the internal reference gene *LRP10*, and in the cell culture experiments to 18S. Relative expression was calculated using the ${2}^{-\Delta {\mathrm{C}}_{\mathrm{t}}}$ method [25].

### Glycerol measurements

Glycerol in media was measured using Free Glycerol Reagent (Sigma Aldrich, St Louis, MO, USA) and Amplex UltraRed (Invitrogen), according to the manufacturers’ instructions. Amplex Ultra Red was diluted 100-fold in Free Glycerol Reagent, mixed with 20 μl of conditioned medium in a 96-well plate, and incubated at room temperature for 15 min. Fluorescence was measured (excitation/emission wavelengths 530 nm / 590 nm) using an Infinite M200 plate reader (Tecan Group, Männedorf, Switzerland).

### Statistical analysis

FSI, lipolysis and lipogenesis measurements were log_10_ transformed before analysis to obtain normally distributed variables. Microarray results from the clinical cohort were analysed by regression in QLUCORE version 3.2 (www.qlucore.com), adjusting for array batch and, as indicated, age. Phenotypes correlating with BMI (i.e. FSI, FPG, insulin-stimulated lipogenesis and lipolysis) were analysed both without and with BMI as covariates, as specified. False discovery rate (FDR) was used to adjust for the analysis of multiple genes. FDR <5% was considered significant. Quantitative RT-PCR results and clinical variable were analysed by regression in JJMP v. 11 (www.jmp.com). Results of in vitro experiments were analysed by paired *t* test.

## Results

### FSI and adipose traits

**Fig. 1:**
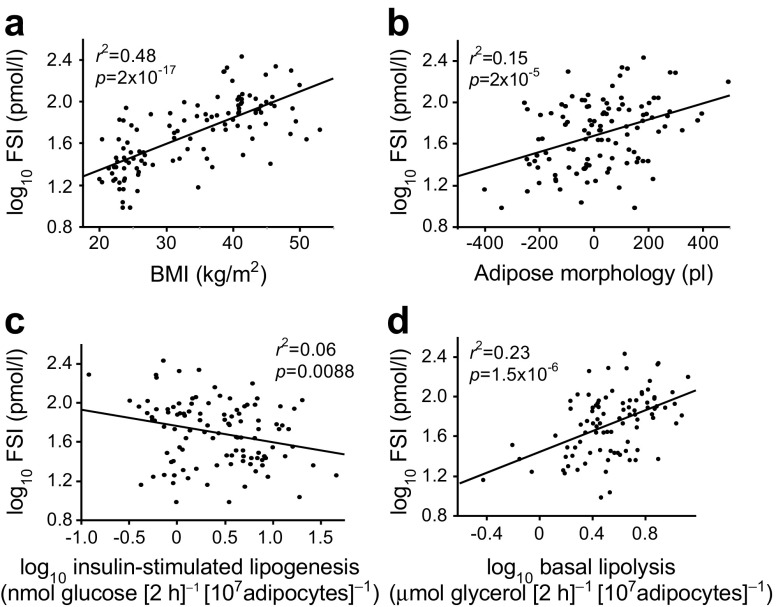
Relationship between FSI and BMI (**a**), adipose morphology (**b**), insulin-stimulated lipogenesis (**c**) and basal lipolysis (**d**). *n* = 114 women. Defined in the ‘WAT experiments’ section of the Methods. Linear regression was used in all analyses

The cohort characteristics are shown in Table 1. The studied women displayed a wide variation in BMI and FSI. As expected, BMI was positively correlated with FSI (*r*^2^ = 0.48, *p* = 2.0 × 10^−17^) (Fig. 1a). BMI was also positively correlated with adipose spontaneous lipolysis (*r*^2^ = 0.15, *p* = 0.0002) and inversely correlated with insulin-stimulated lipogenesis (*r*^2^ = 0.08; *p* = 0.0038) (results not shown). There was no significant association with adipose morphology. FSI was positively correlated with adipose morphology (*r*^2^ = 0.15, *p* = 2.0 × 10^−5^) and spontaneous lipolysis (*r*^2^ = 0.23, *p* = 1.5 × 10^−6^) but was negatively correlated with insulin-stimulated lipogenesis (*r*^2^ = 0.06, *p* = 0.0088) (Fig. 1b–d). Associations between FSI and morphology (*p* = 1.3 × 10^−6^) or spontaneous lipolysis (*p* = 0.0019) remained significant in a multiple regression including BMI as independent variable. There was no influence of age on the examined variables (results not shown).

### Expression of candidate genes for FSI in relation to adipose traits

We next examined whether WAT expression of genes in loci robustly associated with FSI and FSIadjBMI were associated with clinical or adipose phenotypes related to IR. There were 135 protein-coding transcripts in the 17 FSI- and FSIadjBMI-associated loci (ESM Table 1) and expression of 120 of these transcripts were quantified by array. We did not analyse non-coding transcripts since microRNAs were not enriched in the total prepared RNA from WAT, and other non-coding transcripts and RNAs were not represented with probes on the arrays. Expression of 56 genes was associated with BMI (FDR <5%) (Table 2 and ESM Table 2) but only one gene was associated with WHR. The expression of 48 genes was associated with FSI, of which 11 remained nominally significant after adjustment for BMI, whereas 19 genes were associated with FPG, of which two remained nominally significant after adjustment for BMI.

**Table 2:** Adipose expression of candidate genes in loci associated with FSI and their association with clinical and adipose variables

Chr	Trait	SNP	Gene	BMI		FSI		FPG		Basal lipolysis		Insulin-stimulated lipogenesis		WAT morphology	
				*p* value	*R*	*p* value	*R*	*p* value	*R*	*p* value	*R*	*p* value	*R*	*p* value	*R*
1	FSI	rs2820436	*LYPLAL1* ^a^	1.2 × 10^−4b^	–	1.1 × 10^−2b^	–	4.4 × 10^−2^	–	2.6 × 10^−2^	–				
	FSIadjBMI	rs4846565	*EPRS*	1.6 × 10^−3b^	+	1.5 × 10^−5b,c^	+					4.8 × 10^−3b^	–		
			*IARS2*	1.3 × 10^−4b^	–	4.0 × 10^−3b^	–			2.0 × 10^−2^	–				
			*RAB3GAP2*	8.7 × 10^−4b^	–	8.4 × 10^−3b^	–	7.1 × 10^−3b^	–	1.9 × 10^−4b,c^	–			1.0 × 10^−2^	–
2	FSI	rs1530559	*R3HDM1*									1.5 × 10^−5a,c^	+		
			*UBXN4*									9.7 × 10^−a^	–		
			*MCM6*					2.2 × 10^−2^	–	2.4 × 10^−3b,c^	–			3.6 × 10^−2^	–
			*DARS*	3.6 × 10^−12b^	–	3.9 × 10^−8b^	–	2.0 × 10^−3b^	–	9.3 × 10^−4b^	–	6.9 × 10^−4b^	+	3.2 × 10^−2^	–
			*CXCR4*	6.9 × 10^−5b^	+	1.1 × 10^−3b^	+	3.3 × 10^−2^	+	2.2 × 10^−2^	+	2.7 × 10^−2^	–	4.4 × 10^−3^	+
2	FSI	rs10195252	*GRB14* ^a^	1.1 × 10^−2b^	+	2.9 × 10^−2^	*+*					3.5 × 10^−4b,c^	–		
	FSIadjBMI		*CSRNP3*	1.4 × 10^−3b^	+	3.3 × 10^−2^	*+*					4.0 × 10^−2^	–		
			*GALNT3*	1.3 × 10^−2b^	+										
			*TTC21B*	1.3 × 10^−8b^	–	1.0 × 10^−6b^	–	4.2 × 10^−5b,c^	–	7.5 × 10^−4b^	–			8.1 × 10^−3^	–
			*SCN1A*	3.1 × 10^−3b^	–			3.1 × 10^−2^	–	3.6 × 10^−2^	–				
2	FSI	rs2972143	*IRS1* ^a^	1.8 × 10^−7b^	–	2.8 × 10^−5b^	–	2.6 × 10^−3b^	–			3.4 × 10^−4b^	+	3.3 × 10^−2^	–
	FSIadjBMI	rs2943645	*RHBDD1*	1.3 × 10^−2b^	+	3.0 × 10^−3b^	+			1.0 × 10^−2^	+				
			*MFF*	2.3 × 10^−3b^	–										
			*AGFG1*	1.7 × 10^−8b^	+	6.9 × 10^−8b,c^	+	3.9 × 10^−3b^	+	7.9 × 10^−4b^	+	2.7 × 10^−3b^	–	2.5 × 10^−2^	+
3	FSIadjBMI	rs17036328	*PPARG* ^a^	7.1 × 10^−15b^	–	3.9 × 10^−11b,c^	–	3.5 × 10^−3b^	–	8.3 × 10^−6b,c^	–	5.7 × 10^−6b,c^	+	2.8 × 10^−3^	–
			*SYN2*	1.1 × 10^−3b^	–	1.2 × 10^−3b^	–								
			*TSEN2*	2.8 × 10^−5b^	–	1.2 × 10^−3b^	–	4.5 × 10^−2^	–	1.3 × 10^−3b^	–	2.0 × 10^−2^	+	3.0 × 10^−3^	–
			*RAF1*	6.9 × 10^−4b^	+	1.2 × 10^−3b^	+					6.0 × 10^−4b,c^	–		
			*TMEM40*					3.6 × 10^−4b,c^	+						
4	FSIadjBMI	rs3822072	*FAM13A* ^a^	2.2 × 10^−15b^	–	5.6 × 10^−12b,c^	–	6.7 × 10^−4b^	–	5.6 × 10^−7b,c^	–	1.6 × 10^−2^	+	8.2 × 10^−4b^	–
			*GPRIN3*	1.0 × 10^−9b^	+	6.3 × 10^−6b^	+	3.7 × 10^−2^	+			3.5 × 10^−3b^	–		
			*SNCA*	5.8 × 10^−3b^	+	9.6 × 10^−3b^	+	1.4 × 10^−3b^	+	8.2 × 10^−3^	+				
			*MMRN1*	1.4 × 10^−3b^	+	2.2 × 10^−3b^	+	3.0 × 10^−2^	+						
			*CCSER1*	1.1 × 10^−2b^	+							1.6 × 10^−2^	–		
4	FSI	rs9884482	*INTS12*	3.3 × 10^−2^	–	8.8 × 10^−3b^	–			3.5 × 10^−2^	–				
	FSIadjBMI	rs974801	*GSTCD*	7.3 × 10^−3b^	+										
			*TBCK*	1.6 × 10^−2b^	–			2.8 × 10^−2^	–	5.8 × 10^−3b^	–				
			*AIMP1*	4.2 × 10^−5b^	–	7.9 × 10^−4b^	–	1.3x10^−3b^	–	6.2 × 10^−2^	–	2.7 × 10^−2^	+		
4	FSIadjBMI	rs6822892	*FAM198B*	3.3 × 10^−8b^	+	1.4 × 10^−7b,c^	+	3.8 × 10^−3b^	+	2.1 × 10^−2^	+	4.7 × 10^−3b^	–	6.1 × 10^−3^	+
5	FSI	rs4865796	*ARL15* ^a^	1.5 × 10^−5b^	+	1.0 × 10^−4b^	+	2.4 × 10^−2^	+			1.8 × 10^−6b,d^	–	5.2 × 10^−3^	+
	FSIadjBMI		*ITGA2*	2.1 × 10^−5b^	+	5.1 × 10^−5b^	+	1.3 × 10^−2^	+	3.0 × 10^−2^	+	6.4 × 10^−3b,c^	–		
			*MOCS2*	1.1 × 10^−5b^	–	1.3 × 10^−3b^	–			5.2 × 10^−3b^	–			4.6 × 10^−2^	–
			*NDUFS4*	2.0 × 10^−8b^	–	2.4 × 10^−7b^	–			4.0 × 10^−2^	–	2.2 × 10^−4b^	+	5.9 × 10^−5b^	–
5	FSIadjBMI	rs459193	*MAP3K1* ^a^	5.8 × 10^−9b^	+	2.9 × 10^−8b,c^	+	1.0 × 10^−3b^	+	2.9 × 10^−3b^	+	7.2 × 10^−5b^	–	3.7 × 10^−2^	+
			*SKIV2L2*	1.9 × 10^−6b^	–	1.5 × 10^−5b^	–	3.3 × 10^−3^	–	5.5 × 10^−4b^	–			1.4 × 10^−2^	–
6	FSIadjBMI	rs6912327	*UHRF1BP1* ^a^											1.1 × 10^−2^	–
			*NUDT3*	2.9 × 10^−5b^	+	2.0 × 10^−8b,c^	+	1.4 × 10^−3b^	+	8.9 × 10^−3^	+	2.6 × 10^−2^	–		
			*RPS10*			1.0 × 10^−2b^	–							4.2 × 10^−3^	–
			*C6orf106*	1.2 × 10^−5b^	+	3.4 × 10^−4b^	+	3.2 × 10^−2^	+	2.1 × 10^−3b^	+			3.1 × 10^−2^	+
			*TCP11*	6.0 × 10^−3b^	–	1.8 × 10^−2b^	–								
			*SCUBE3*			2.4 × 10^−3b,c^	–								
6	FSI	rs2745353	*RNF146*	2.6 × 10^−6b^	–	8.7 × 10^−4b^	–	6.2 × 10^−3b^	–	3.0 × 10^−3b^	–			8.6 × 10^−3^	–
			*ECHDC1*	2.5 × 10^−2^	–							1.7 × 10^−3a^	+		
			*THEMIS*	5.1 × 10^−5b^	+	7.0 × 10^−5b^	+	7.5 × 10^−3b^	+			5.6 × 10^−3b^	–		
7	FSI	rs1167800	*GTF2I*	8.5 × 10^−6b^	–	9.0 × 10^−4b^	–	3.2 × 10^−2^	–	2.4 × 10^−4b^	–				
			*NCF1*	5.2 × 10^−4b^	+	2.0 × 10^−3b^	+	2.9 × 10^−3b^	+	1.8 × 10^−2^	+				
			*STAG3L2*	2.3 × 10^−4b^	–	2.0 × 10^−2b^	–					3.3 × 10^−4b^	+	1.3 × 10^−2^	–
			*GATSL2*	4.3 × 10^−9b^	+	2.5 × 10^−10b,c^	+			3.8 × 10^−3b^	+	1.7 × 10^−3b^	–	2.1 × 10^−3^	+
			*SPDYE8P*	3.5 × 10^−3b^	–	2.4 × 10^−4b,c^	–	1.0 × 10^−3b^	–						
			*TRIM73*			1.5 × 10^−2b^	–								
			*POM121C*	1.1 × 10^−3b^	–	2.0 × 10^−2b^	–					3.4 × 10^−3b^	+	2.6 × 10^−2^	–
			*PMS2P3*							8.0 × 10^−3^	–			4.8 × 10^−2^	–
8	FSIa	rs983309	*PPP1R3B* ^a^									2.9 × 10^−3b^	–		
	FSIadjBMI	rs2126259	*CLDN23*	4.9 × 10^−3b^	–	1.0 × 10^−2b^	–	2.9 × 10^−3b^	–	1.2 × 10^−2^	–				
			*MFHAS1*	3.0 × 10^−11b^	+	5.1 × 10^−6b^	+			3.7 × 10^−2^	+	2.4 × 10^−4b^	–		
			*ERI1*	4.1 × 10^−5b^	+	1.4 × 10^−3b^	+					2.1 × 10^−2^	–		
			*TNKS*	1.1 × 10^−2b^	–					6.2 × 10^−2^	–				
10	FSI	rs7903146	*TCF7L2* ^a^	1.2 × 10^−3b^	–	4.2 × 10^−2^	–	2.1 × 10^−2^	–	2.4 × 10^−2^	–			3.6 × 10^−3^	–
			*ABLIM1*	1.7 × 10^−5b^	+	1.2 × 10^−3b^	+	3.3 × 10^−2^	+	3.1 × 10^−2^	+	2.4 × 10^−6b^	–		
16	FSI	rs1421085	*FTO* ^*c*^									7.2 × 10^−3b^	+		
			*RBL2*	2.7 × 10^−5b^	–	4.0 × 10^−2^	–	3.3 × 10^−2^	–	2.8 × 10^−4b^	–			9.8 × 10^−3^	–
			*RPGRIP1L*	2.4 × 10^−5b^	+	1.5 × 10^−6b,c^	+			3.4 × 10^−2^	+				
			*IRX3*	1.0 × 10^−3b^	–	9.4 × 10^−3b^	–	1.1 × 10^−2^	–						
19	FSI	rs731839	*PEPD* ^a^	1.6 × 10^−6b^	+	1.5 × 10^−3b^	+	2.4 × 10^−2^	+	1.3 × 10^−2^	+				
	FSIadjBMI		*KCTD15*	7.1 × 10^−3b^	+										

Microarray results from the clinical cohort were analysed by regression in QLUCORE, adjusting for array batch, and for WAT morphology, adjusting for age. Only results with *p* < 0.05 are shown

^a^Original candidate gene from Scott et al [11]

^b^FDR <5%

^c^Nominally significant after adjustment for BMI in QLUCORE

*R*, regression coefficient, which can be either positive (+) or negative (−)

For WAT variables, 24 genes were associated with insulin-stimulated lipogenesis (FDR <5%); five of these genes remained nominally significant after adjustment for BMI. Expression of 17 genes was associated with spontaneous lipolysis and four remained significant after adjusting for BMI. Only two genes were associated with adipose morphology (Table 2, ESM Table 2). Expression of four genes was correlated with age (i.e. *GSTCD*, *IRS1*, *SCN3A* and *TIMP4*); however, adjustment for age did not affect the relationship between expression of these genes and FSI or adipose phenotypes (results not shown). Expression of three genes whose expression associated with FSI was validated by quantitative RT-PCR using RNA from subcutaneous WAT obtained from a previously examined cohort of 55 women (ESM Table 3) [26]. Results were directionally consistent between microarray and quantitative RT-PCR for all three genes and analysed phenotypes, except for the association between insulin-stimulated lipogenesis and *ARL15* expression levels.

### Network analysis

The 48 genes whose expression related to FSI, 24 genes related to insulin-stimulated lipogenesis and 17 genes related to lipolysis were analysed in Ingenuity Pathway Analysis. The genes associated with insulin-stimulated lipogenesis were enriched in several signalling pathways, including activation of NF-κB and MAPK signalling (ESM Table 4). These results were related to altered expression of *PPARG*, *IRS1*, *ITGA2*, *RAF1* and *MAP3K1*. Interestingly, the same signalling pathways, including NF-κB activation, were also enriched among FSI-associated genes; the genes mentioned above also contributed to the results for FSI (ESM Table 5). The lipolysis-associated genes were linked to a different set of pathways (results not shown). Network analysis provide little evidence of connection between genes associated with insulin-stimulated lipogenesis or lipolysis (ESM Figs 1, 2). *PPARG* was the only FSI-associated gene with several indirect connections to other FSI-associated genes, pointing to a weak functional connection between FSI-associated genes (ESM Fig. 3). Bearing this in mind, we continued to evaluate the SNPs and candidate genes for FSI one at a time.

### Linking candidate genes for FSI to WAT function by eQTL analysis and siRNA knockdown

To further link candidate genes from GWAS to WAT function, we used in silico analysis to determine whether tag-SNPs for each of the 17 FSI- or FSIadjBMI-associated loci (ESM Table 1) comprised *cis* eQTLs. According to GTEx, eight genes in four different genetic loci demonstrate genotype-specific expression (Table 3). At three of these loci, WAT gene expression associated with FSI and adipose phenotypes (FDR <5%). At the fourth locus, *UHRF1BP1* was nominally associated with adipose morphology. According to the SNP annotation tool SNPnexus, three of the SNPs are intronic and the fourth intergenic (Table 3). None of the SNPs has any regulatory function ascribed to them.

**Table 3:** SNPs containing *cis* eQTLs

Chr	SNP	Locus for FSI GWAS		eQTL^a^				FSI^b^		Lipolysis^b^		Insulin-stimulated lipogenesis^b^		WAT morphology^b^	
			Effect allele	Gene	Effect allele	*p* value	Effect size	*p* value	*R*	*p* value	*R*	*p* value	*R*	*p* value	*R*
2	rs2972143	Intergenic	G	*IRS1*	G	8.0 × 10^−8^	−0.29	2.8 × 10^−5c^	–			3.4 × 10^−4c^	+	3.3 × 10^−2^	–
4	rs3822072	Intronic	A	*FAM13A*	G	4.0 × 10^−6^	0.24	5.6 × 10^−12c^	–	5.6 × 10^−7c^	–	1.6 × 10^−2^	+	8.2 × 10^−4c^	–
6	rs6912327	Intronic	T	*UHRF1BP1*	C	2.0 × 10^−25^	0.51							1.1 × 10^−2^	–
			T	*SNRPC*	C	3.3 × 10^−10^	−0.28								
7	rs1167800	Intronic^d^	A	*STAG3L1*	A	3.3 × 10^−10^	−0.28								
			A	*TRIM73*	A	5.2 × 10^−8^	−0.34	1.5 × 10^−2c^	–						
			A	*POM121C*	A	3.6 × 10^−7^	−0.22	2.0 × 10^−2c^	–			3.4 × 10^−3c^	+	2.6 × 10^−2^	–
			A	*PMS2P3*	A	6.1 × 10^−11^	−0.41			8.0 × 10^−3^	–			4.8 × 10^−2^	–

^a^SAT eQTL according to GTEx portal

^b^Microarray results from the clinical cohort were analysed by regression in QLUCORE, adjusting for array batch, and for WAT morphology, adjusting for age. Only results with *p* < 0.05 are shown

^c^FDR <5%

^d^Intronic in the *HIP1* gene

*R*, regression coefficient, which can be either positive (+) or negative (−)

**Fig. 2:**
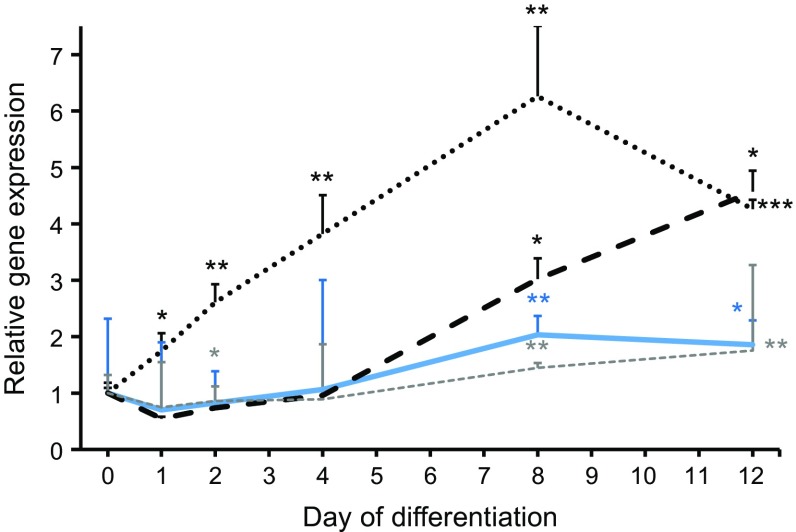
Gene expression of *FAM13A*, *POM121C*, *SNRPC* and *UHRF1BP1* were monitored using quantitative RT-PCR during differentiation of hMSCs to adipocytes in vitro from start of differentiation (day 0) until day 12. *POM121C* (solid blue line), *FAM13A* (dotted black line), *UHRF1BP1* (dashed black line) and *SNRPC* (dashed grey line). Results were analysed using the paired *t* test and are presented as relative fold change+SD vs day 0. *p < 0.05, **p < 0.01 and ***p < 0.001 vs day 0

**Fig. 3:**
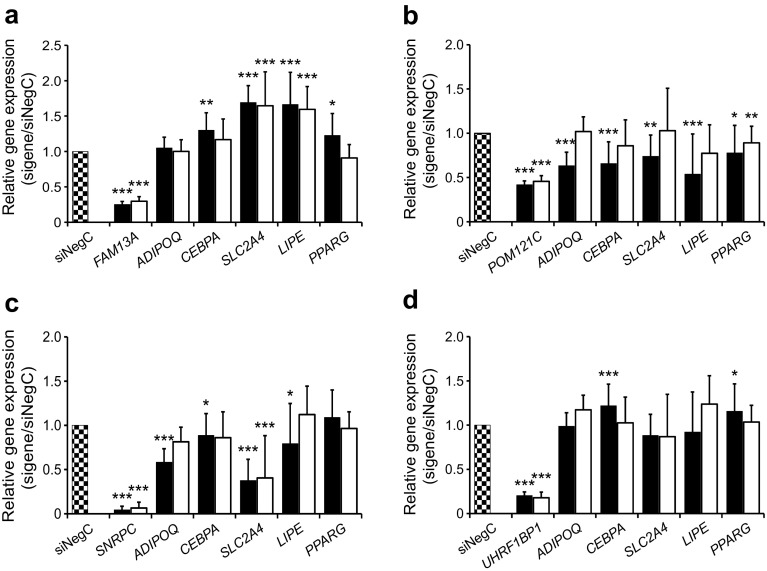
Expression of *FAM13A* (**a**), *POM121C* (**b**), *SNRPC* (**c**) and *UHRF1BP1* (**d**) was knocked down using siRNA in hMSCs in vitro at day 4 of differentiation until day 7 and 12 of differentiation, upon which the expression of target and *ADIPOQ*, *CEBPA*, *SLC2A4*, *LIPE* and *PPARG* was monitored. We have performed three biological experiments with 3–4 technical replicates in each experiment; *n* = 11 technical replicates for NegC; *n* = 12 technical replicates for target genes. Results were analysed using the paired *t* test and are presented as relative fold change±SD vs negative control (NegC) at each time point during differentiation. Black bars, day 7; white bars, day 12. *p < 0.05, **p < 0.01 and ***p < 0.001 vs NegC

Four genes comprising eQTLs (*FAM13A*, *UHRF1BP1*, *POM121C* and *SNPRC*) were taken forward for functional evaluation in hMSCs to determine possible impact on adipocyte function. We first evaluated expression of the four genes during differentiation of hMSCs. The expression of *FAM13A* increased directly after induction of differentiation, whereas the expression of *POM121C*, *UHRF1BP1* and *SNRPC* displayed detectable increases only at day 8 (Fig. 2). Based on these data, the genes were knocked down using siRNA in the hMSCs at day 4, corresponding to an early stage of differentiation. Three and eight days post-transfection (i.e. day 7 and 12 of differentiation), this resulted in a decreased expression of *FAM13A*, *UHRF1BP1* and *SNPRC* by 70–95% and decreased expression of *POM121C* by 60% (Fig. 3).

**Fig. 4:**
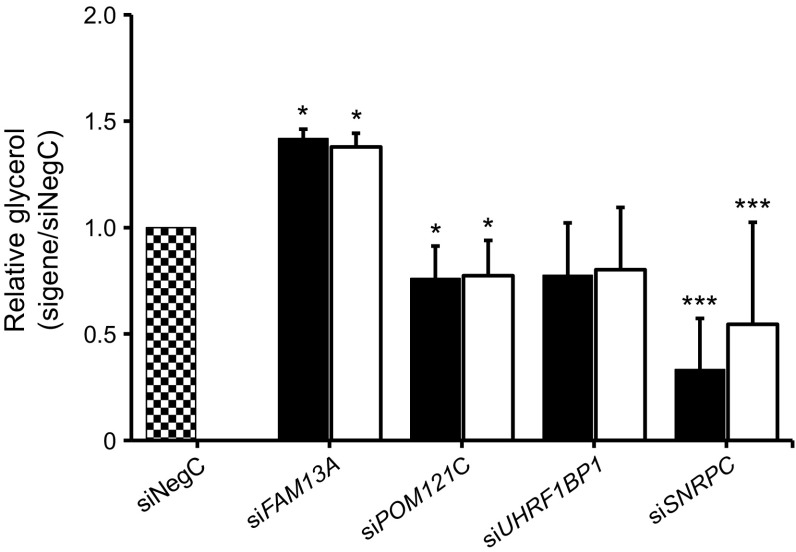
Expression of *FAM13A*, *POM121C*, *SNRPC* and *UHRF1BP1* was knocked down using siRNA in hMSCs in vitro and glycerol levels in conditional medium were evaluated. We have performed 3 biological experiments with 3–4 technical replicates in each experiment; *n* = 11 technical replicates for NegC; *n* = 12 technical replicates for target genes. Results were analysed using the paired *t* test and are presented as relative fold change±SD vs non-targeting siRNA pool (siNegC). Black bars, day 7; white bars, day 12. *p < 0.05 and ***p < 0.001 vs NegC

Expression levels of adipocyte-enriched genes central to adipogenesis (*PPARG*, *CEBPA*), lipolysis (*LIPE*) and insulin sensitivity (*SLC2A4*, *ADIPOQ*) were measured in each knockdown experiment. Knockdown of *FAM13A* increased expression of *LIPE*, *SLC2A4*, *PPARG* and *CEBPA* (Fig. 3a). We also evaluated levels of glycerol in the conditioned medium as a marker for lipolysis. Glycerol levels increased significantly, by approximately 1.5-fold, indicating an increased lipolysis (Fig. 4). As *FAM13A* expression increased from the very beginning of differentiation, we also knocked down *FAM13A* one day prior to induction of differentiation (day −1) and this had a similar effect on adipocyte-specific gene expression (results not shown). *POM121C* knockdown resulted in significantly reduced expression of all investigated genes (Fig. 3b) and reduced glycerol release (Fig. 4). Knockdown of *SNRPC* resulted in strongly reduced expression of *ADIPOQ* and *SLC2A4*, as well as modestly decreased expression of *LIPE* and *CEBPA* (Fig. 3c). The glycerol level in the medium was significantly reduced, which is in line with gene expression data (Fig. 4). *UHRF1BP1* knockdown resulted in a temporary and modest increase in the expression of *PPARG* and *CEBPA*, with no effect on glycerol release in medium. Four eQTL genes were excluded from the functional evaluation in vitro: *IRS1* because its function is already defined; *PMS2P3*, *TRIM73* and *STAG3L1* since commercial siRNAs reagents were unavailable, or because the genes were expressed at low levels in adipocytes or below the detection threshold in WAT.

## Discussion

To explore the role of WAT in the genetic predisposition to systemic IR we have analysed adipose expression of 120 candidate genes in genetic loci associated with FSI and FSIadjBMI. We report that adipose expression of a surprisingly high number of these genes (48; 40%) associate with FSI and with fat cell insulin sensitivity or lipolysis (35; 30%). These findings give support to the notion that WAT is an important organ mediating the effects of genetic variants on FSI. Furthermore, functional analysis in vitro by siRNA-mediated knockdown highlighted *FAM13A* and *POM121C* as potential causal links between SNPs and IR at specified GWAS loci. None of these genes have to our knowledge previously been implicated in WAT function in humans.

At one chromosome 4 locus (rs3822072), the FSI-associated allele is associated with lower *FAM13A* expression. Consistent with a protective role of *FAM13A* on IR, we show that *FAM13A* expression is associated with a beneficial metabolic profile, including decreased WAT lipolysis, and that *FAM13A* knockdown increases lipolysis and expression of *LIPE*, which is a rate-limiting enzyme in lipolysis [27]. We previously showed that basal lipolytic activity is a strong determination of insulin sensitivity [20]. The increased expression of other genes with central functions in adipogenesis and adipocytes (e.g. *PPARG*, *CEBPA* and *SLC2A4*) following *FAM13A* knockdown indicate that *FAM13A* has the potential to inhibit adipogenesis; however, the clinical relevance of this finding is unclear. The precise molecular mechanisms linking *FAM13A* to lipolysis are unknown. *FAM13A* has previously been reported to induce fatty acid oxidation, autophagia via activation of Akt and β-catenin degradation in various cell types [28–30]. Akt controls lipolysis [31] and could hypothetically be involved in *FAM13A* inhibition of lipolysis; however, detailed mechanistic studies are beyond the scope of this investigation.

At the chromosome 7 locus (rs1167800), the FSI-associated allele is associated with lower *POM121C* expression. Consistent with an insulin-sensitising function, *POM121C* expression is positively associated with systemic and adipocyte insulin sensitivity and with adipose hyperplasia. Adipose hypertrophy has previously been linked to impaired adipogenesis and IR [8]. *POM121C* knockdown early in the adipogenesis caused decreased expression of all adipocyte-specific markers suggesting that *POM121C* is necessary for adipocyte differentiation. We observed reduced glycerol release following *POM121C* knockdown. This is probably secondary to impaired adipogenesis (i.e. without functional adipocytes there is no lipolysis). *POM121C* encodes a nucleoporin and forms an important component of the nuclear pore complexes [32]; it has to our knowledge previously not been implicated in human metabolic disease.

At one chromosome 6 locus (rs6912327), the effects observed after siRNA-mediated knockdown of *SNRPC* and *UHRF1BP1* did not suggest that these genes underlie genetic control of FSI at this locus. Thus, the FSI-associated allele is associated with higher *SNPRPC* expression, suggesting that *SNRPC* contributes to metabolic disease. However, *SNPRPC* knockdown reduced expression of *ADIPOQ*, encoding the insulin-sensitising hormone adiponectin, and *SLC2A4*, encoding the glucose transporter GLUT4; these findings are consistent with an insulin-sensitising function of *SNRPC*. Lipolysis was also reduced, potentially secondary to impaired lipid accumulation. Knockdown of *UHRF1BP1* had marginal effects on adipocyte markers, suggesting that *UHRF1BP* is not a causative gene for FSI. Of note, expression of neither *UHRF1BP* nor *SNPRPC* was associated with WAT variables with FDR <5%.

The present study has several limitations. The investigated genomic regions are based on distance and not linkage disequilibrium (LD) patterns and we cannot exclude that genes with more distal *cis*-eQTL effects might play a role in WAT function. To our knowledge, however, no reliable LD data are available for this Swedish population. Furthermore, in Ensemble (www.ensemble.org, accessed 22 November 2017) we did not find any non-synonymous, or otherwise likely functional, SNPs in high LD (*r*^2^ ≥ 0.9) with rs3822072 or rs1167800. Another limitation is that we studied women only and our results cannot be generalised to the entire population. The analysis was limited to abdominal subcutaneous WAT and the results, thus, cannot be generalised to other WAT depots. The relative importance of subcutaneous vs visceral WAT for IR is debatable. It is notable that removal of the greater omentum in addition to bariatric surgery is not associated with metabolic benefits after long-term follow-up, pointing to the limited importance of visceral WAT for metabolic disease in this population [33].

Only a few of the candidate genes for FSI shown in Table 2 were taken forward for functional evaluation. Beyond *FAM13A* and *POM121C*, however, a few of the genes in Table 2 have functions ascribed to them which support the notion that they might influence the metabolic function of WAT and systemic IR: *PPARG* and *IRS1* control adipogenesis and insulin signalling, respectively [34, 35]; *MAP3K1* and *TCF7L2* are involved in Wnt signalling [36, 37], which inhibits adipogenesis; *GRB14* is directly involved in insulin signalling [38] and *ARL15* is encoded in a major genetic locus controlling adiponectin levels [39]. However, information about function is lacking for most of the genes in Table 2, making it difficult to define networks based on functional connectivity between the genes.

Many genetic variants seem to have pleiotropic effects on metabolic traits [11]. The genetic locus on chromosome 4 harbouring *FAM13A* has previously been linked to body fat distribution [40], and the chromosome 7 locus harbouring *POM121C* has been linked to BMI [41]. Thus, it is possible that the impact of these genes on lipolysis and adipogenesis is important for development of obesity as well. At the same time, the observation that several of the examined genes are associated with more than one studied phenotype emphasizes the functional connection between studied phenotypes (e.g. large fat cells are insulin resistant and display enhanced spontaneous lipolysis [8]).

In conclusion, gene expression and adipocyte functional studies support the notion that *FAM13A* and *POM121C* control adipocyte lipolysis and adipogenesis, respectively, and might thereby be involved in genetic control of systemic insulin sensitivity and, possibly, fat accumulation.

## Electronic supplementary material

ESM Figs (PDF 375 kb)

ESM Tables (XLSX 50 kb)

## Data Availability

The datasets generated and/or analysed during the current study are available from the corresponding author on reasonable request.

## Abbreviations

eQTL: Expression quantitative trait locus
FDR: False discovery rate
FPG: Fasting plasma glucose
FSI: Fasting serum insulin
FSIadjBMI: FSI adjusted for BMI
GWAS: Genome-wide association study
hMSC: Human mesenchymal stem cell
IR: Insulin resistance
LD: Linkage disequilibrium
siRNA: Small interfering RNA
WAT: White adipose tissue
WHRadjBMI: WHR adjusted for BMI
