# Supplementary material for: FAM13A and POM121C are candidate genes for fasting insulin: functional follow-up analysis of a genome-wide association study
Source: Diabetologia. 2018 Feb 27;61(5):1112–23. doi: 10.1007/s00125-018-4572-8 (PMC6448992; doi:10.1007/s00125-018-4572-8)
Supplement: Supplementary file 1 — (PDF 375 kb) [file 125_2018_4572_MOESM1_ESM.pdf]

ESM Fig. 1

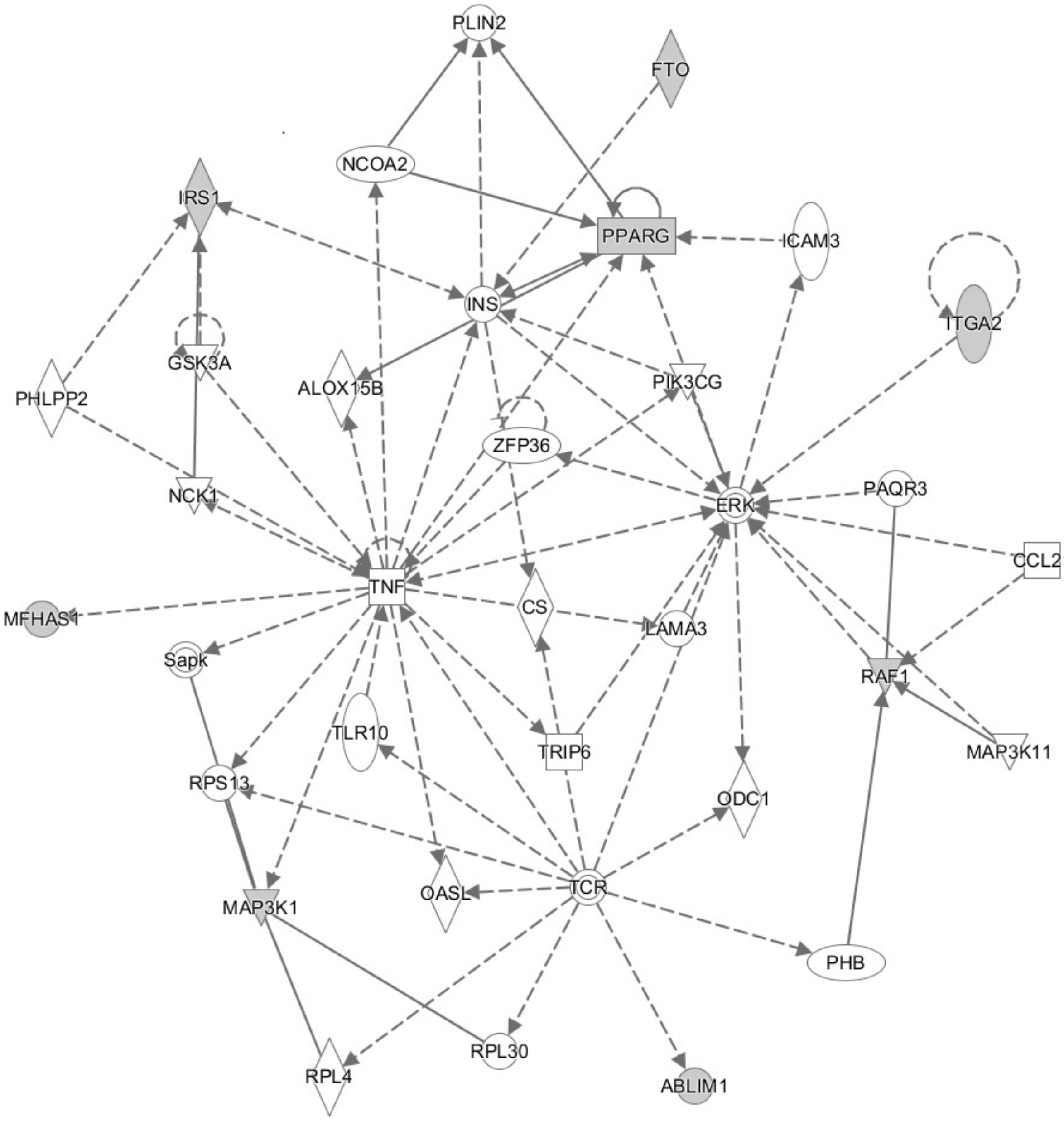

ESM Fig. 1. Ingenuity Pathway Analysis (IPA) was used to construct a network based on 24 genes associated with insulin-stimulated Lipogenesis. The analysis was based on default settings in IPA. Edges indicate relationships experimentally observed in humans or predicted with high score. Arrows indicate direction of relationship. Nodes represent genes. Candidate genes associated with adipose phenotype of interest are shaded grey in the pathway. Pathway molecules that are not in our dataset are white. The shapes and positions of the molecules in the pathway define gene type and cellular location, respectively.

ESM Fig. 2

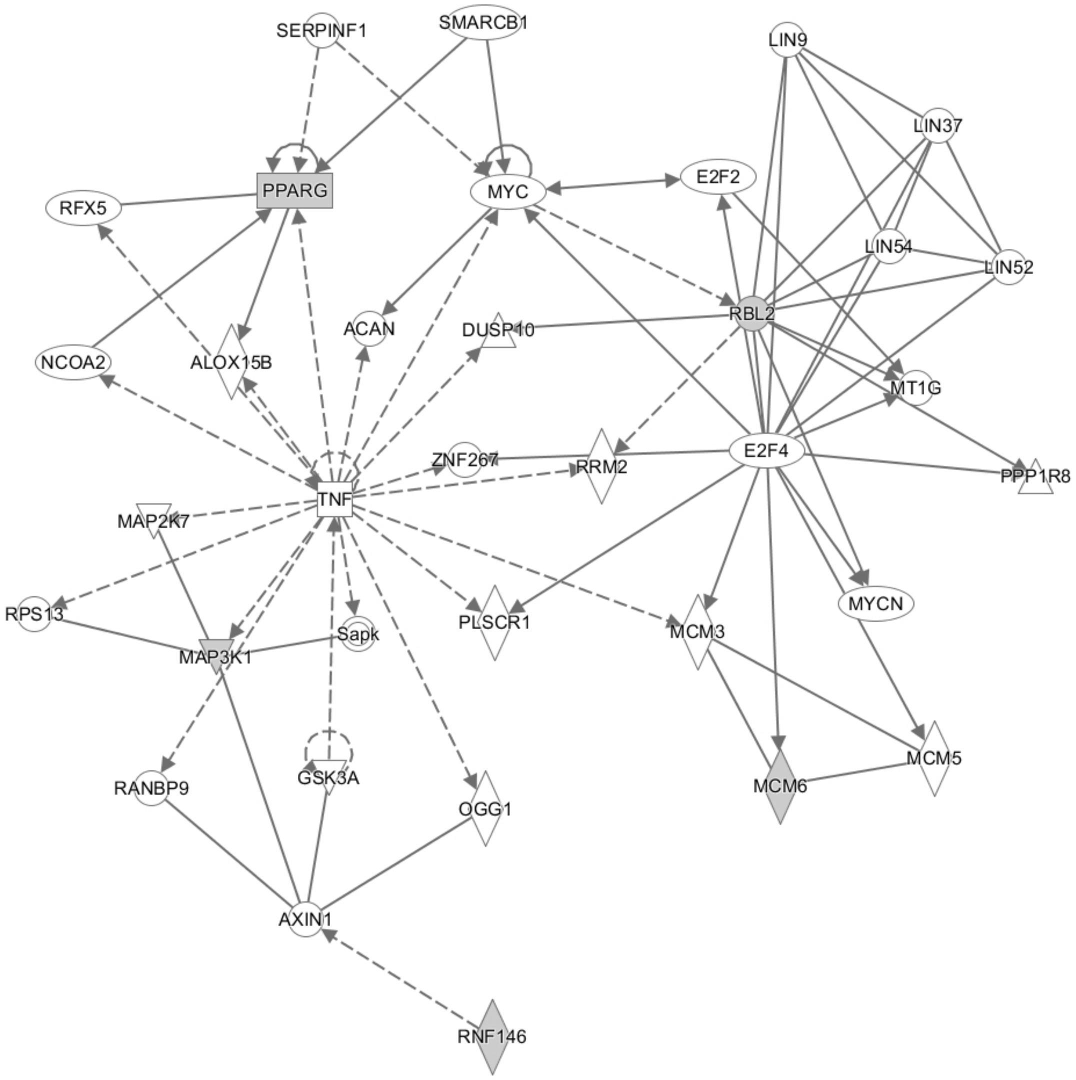

© 2000-2017 QIAGEN. All rights reserved.

ESM Fig. 2. Ingenuity Pathway Analysis was used to construct a network based on 17 genes associated with lipolysis.. See ESM Fig 1 for details of the analysis.

ESM Fig. 3

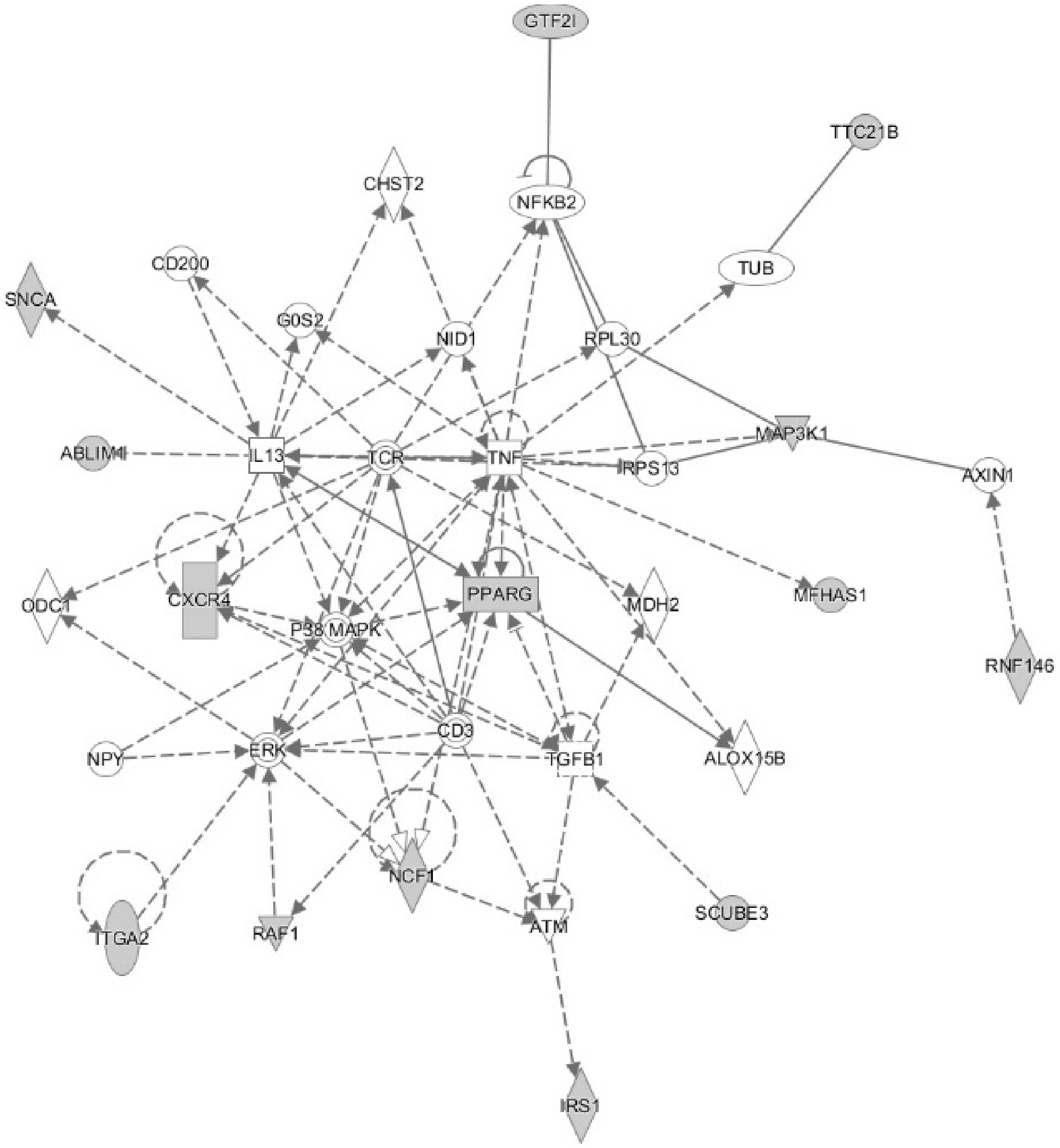

ESM Fig. 3. Ingenuity Pathway Analysis was used to construct a network based on 48 genes associated with FI. See ESM Fig 1 for details of the analysis.
